# Revision Total Hip Arthroplasty in Jehovah’s Witnesses at a Public Hospital: Practical Recommendations for a Low-Resource Setting

**DOI:** 10.7759/cureus.15761

**Published:** 2021-06-19

**Authors:** Marlon M Mencia, Allan Beharry, Pedro P Hernandez Cruz

**Affiliations:** 1 Department of Clinical Surgical Sciences, The University of the West Indies, St. Augustine, TTO; 2 Department of Surgery, Port of Spain General Hospital, Port of Spain, TTO

**Keywords:** jehovah’s witnesses, revision hip arthroplasty, blood transfusion, trauma, low-resource setting

## Abstract

Revision total hip arthroplasty (THA) is a major reconstructive procedure traditionally associated with significant blood loss. Jehovah’s Witnesses (JW) do not accept blood or blood product transfusions because of their religious beliefs. When confronted with a JW patient requiring a complex arthroplasty procedure, surgeons face moral and ethical questions and may be reluctant to perform surgery. A successful outcome depends on several factors including surgical and anesthetic expertise, a range of revision implants, and a multimodal blood management protocol. While these resources are readily available in a developed country, in many of the developing Caribbean islands, the healthcare system is underfunded and under-resourced.

Here, we describe our experience performing a revision THA on a JW patient in the Caribbean. Through this case report, we aim to illustrate our approach to blood management by exploring the fundamental elements that were employed in a low-resource setting. We believe that the extrapolation of these crucial principles to the broader category of primary arthroplasty in the general population can be used to reduce the rate of blood transfusion, increase access to surgery, and improve outcomes.

## Introduction

Total hip arthroplasty (THA) is an effective surgical procedure that has improved the lives of millions of patients with end-stage arthritis [1]. Its use is predicted to increase in line with the global burden of osteoarthritis [2]. However, several questions surround the survivorship of the implant. A recent meta-analysis reported that surgeons could expect a hip replacement to last 15 years in 89.4% of patients [1]. With joint replacement progressively being offered to younger patients, the revision burden is expected to increase by 137% over the next two decades [2].

For a surgeon, accepting a Jehovah’s Witnesses (JW) patient to undergo a revision THA poses several clinical challenges and specific ethical and philosophical questions. JW are members of a Christian denomination whose refusal to accept blood or blood products is based on their literal interpretation of the Bible [3]. In this situation, the surgeon is faced with respecting the patient’s religious beliefs against the need to provide safe surgery. Logically, such complex decision-making is best accomplished by a shared decision model involving the key stakeholders.

Historically, revision THA has been associated with a high rate of blood transfusion [4]. However, recent studies have documented a reduction in blood transfusion rates [4]. Bloodless surgery protocols, developed in part to meet the needs of the JW population, are part of any contemporary arthroplasty program [5]. While several studies have reported successful outcomes of primary THA in the JW population, there remains a paucity of data on patients who undergo revision THA [6-10].

We present the case of a JW patient who underwent a successful revision THA in a resource-limited public hospital in the Caribbean. This report describes the practical application of evidence-based guidelines to reduce the need for blood transfusion in a low-resource setting.

## Case presentation

**Figure 1:**
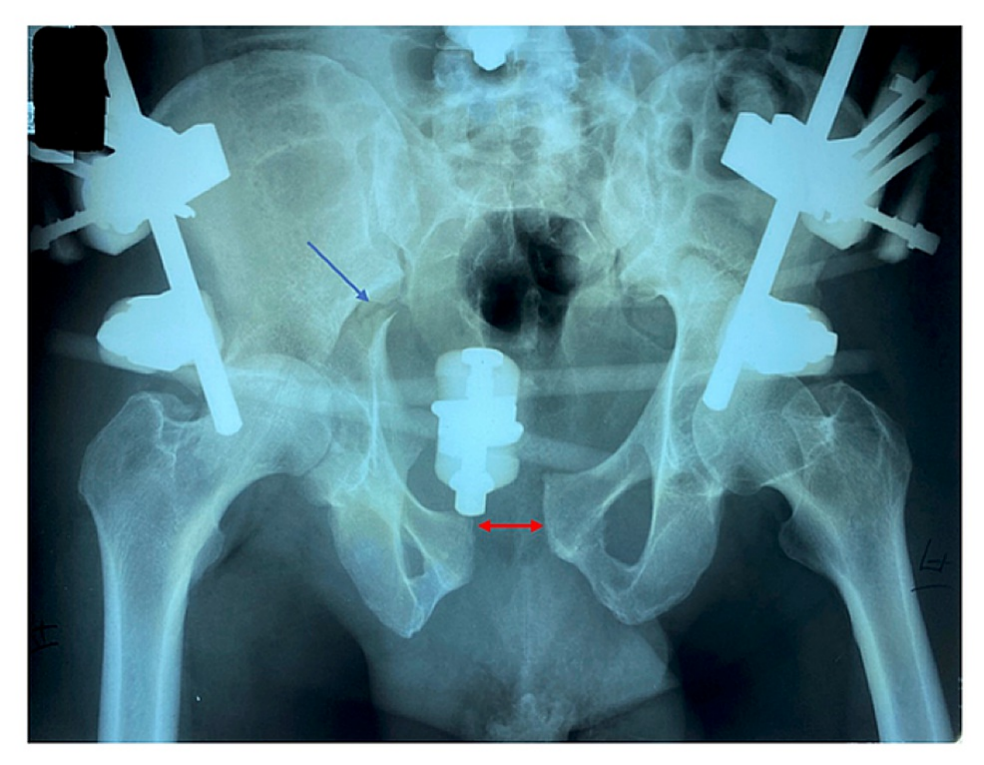
Radiograph of the pelvis with the external fixator applied. The blue arrow shows the transverse acetabular fracture extending into the sacroiliac joint, and the red arrow shows the widening of the pubic symphysis).

A 61-year-old male JW presented to the clinic in 2014 four years after undergoing a complex right THA for post-traumatic osteoarthritis. His initial injury was a combined acetabular and pelvic ring injury (transverse and posterior acetabular wall with an anteroposterior compression (APC II) of the pelvis) sustained in a motor vehicle accident (Figure 1).

**Figure 2:**
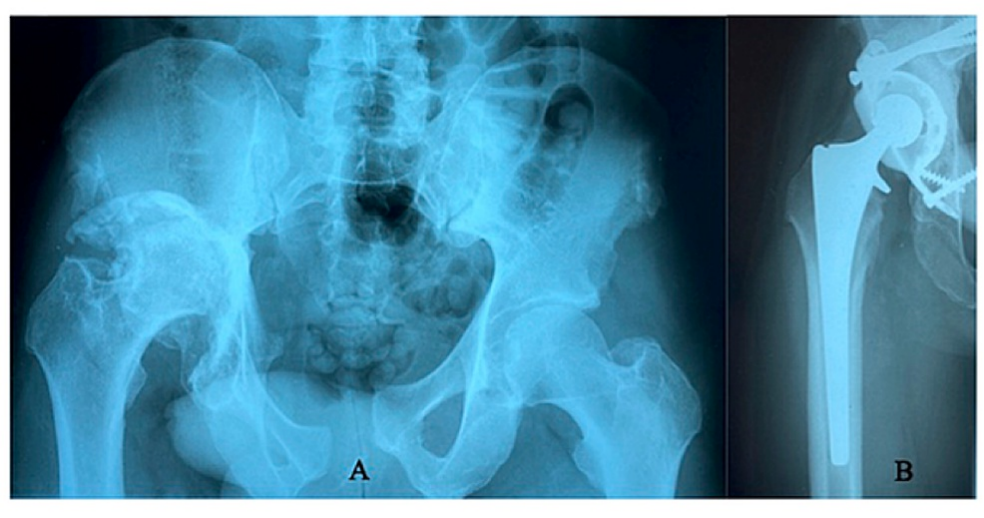
(A) Radiograph showing advanced post-traumatic osteoarthritis of the right hip with superior migration of the hip center and massive acetabular widening. (B) Postoperative radiograph showing good positioning of the hip replacement using a Burch-Schneider reinforcement cage and autograft to reconstruct the acetabulum.

Our team performed his primary arthroplasty using a cage to reconstruct the deficient posterior acetabular wall in combination with ultra-high-molecular-weight polyethylene cemented cup and an uncemented femoral stem. The patient tolerated the operation very well and returned to his work as a taxi driver eight weeks later (Figure 2).

**Figure 3:**
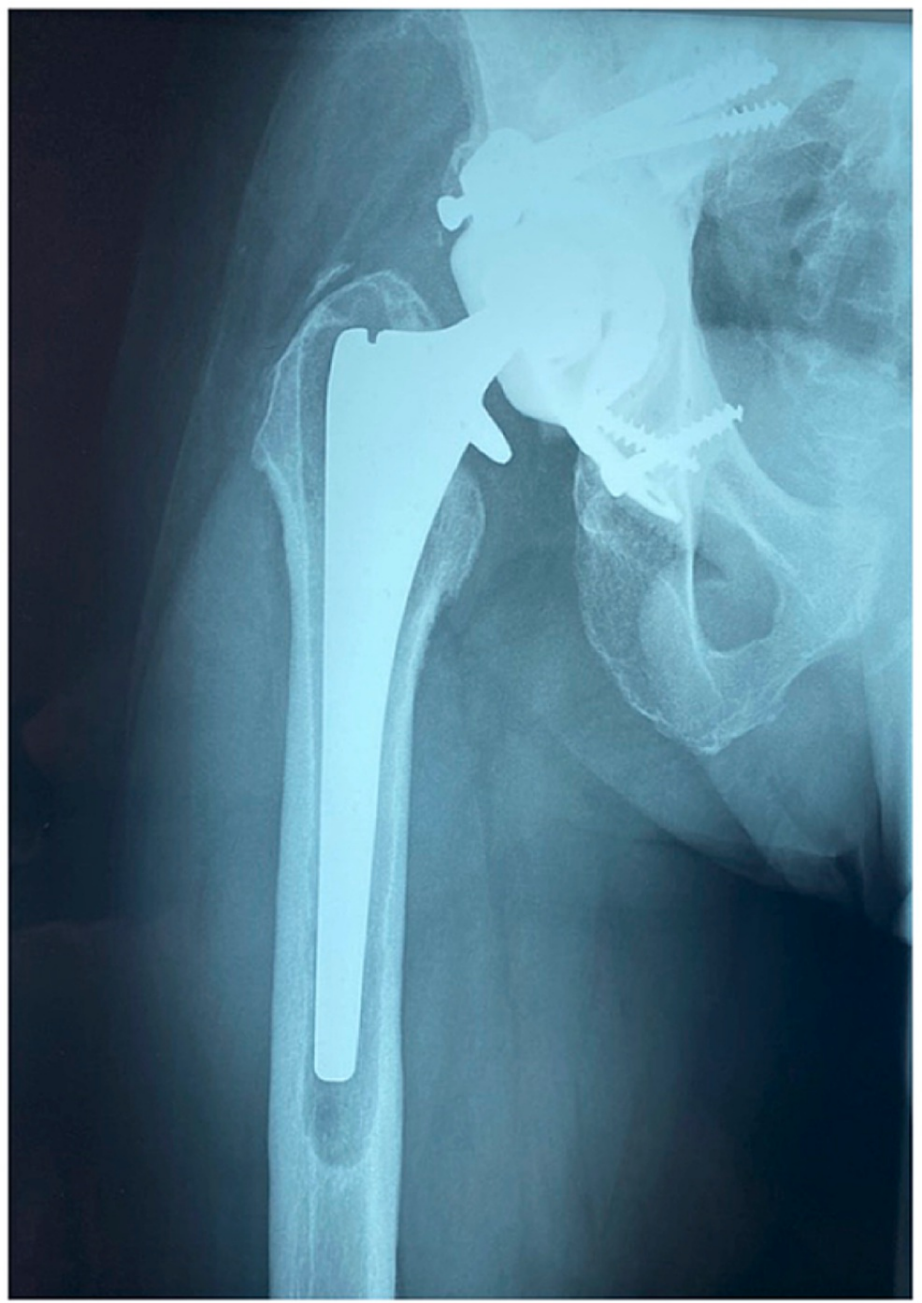
Radiograph of the hip replacement showing evidence of loosening of the femoral stem with lucent lines in Gruen zones 1-7.

On the current presentation, the patient complained of severe thigh pain, and radiographs showed evidence of femoral stem loosening (Figure 3).

**Figure 4:**
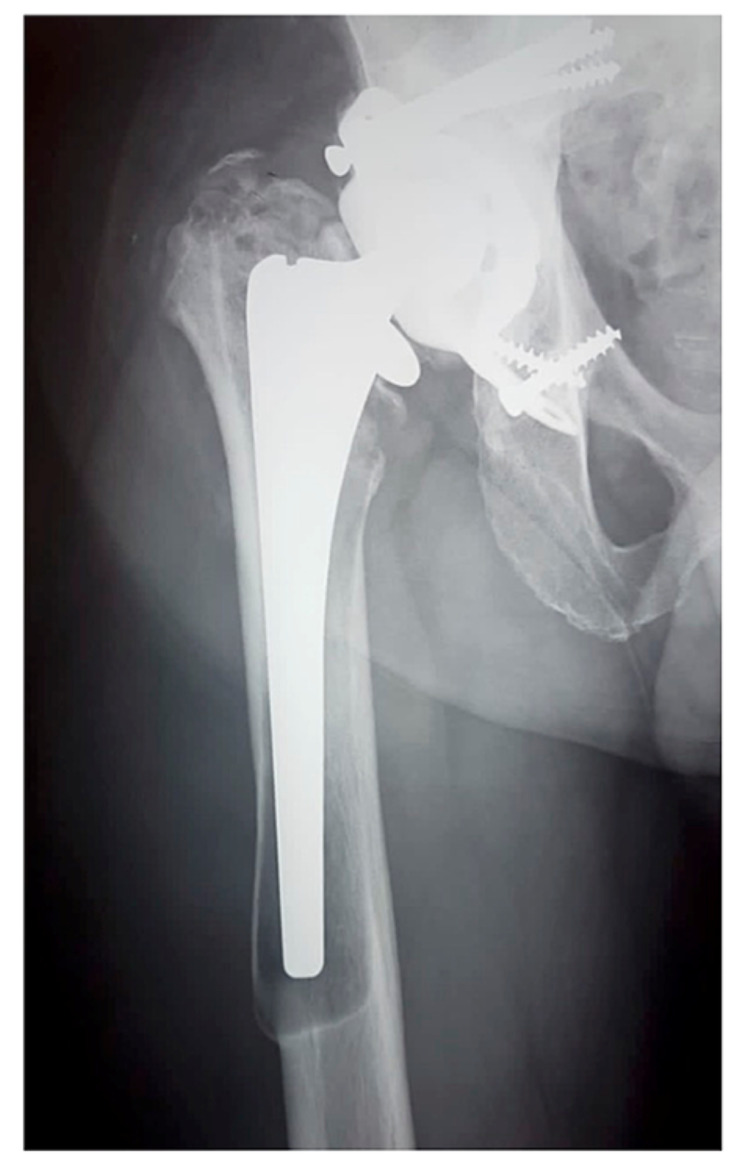
Radiograph showing progressive loosening of the femoral stem with distal migration and osteolysis at the tip of the stem with the risk of impending cortical perforation.

Regrettably, fundamental institutional inadequacies delayed his proposed operation for seven years, by which time there was evidence of progressive loosening (Figure 4).

In preparation for surgery, he was cleared by the medical team. His blood investigations were normal (hemoglobulin of 14.1 g/dL, serum creatinine of 0.96 mg/dL, C-reactive protein of 7.8 mg/dL, and erythrocyte sedimentation rate of 12 mm/hour). As part of our shared decision-making and informed consent process, we thoroughly counseled the patient about the risks of the surgery and specifically about the possibility that, in the presence of uncontrollable hemorrhage, the operation may need to be abandoned and completed in stages.

In the operation theater, under general anesthesia, we used a modified Hardinge approach to access the hip joint. We exercised extreme care in handling the soft tissues and avoided excessive tissue stripping while developing the surgical planes. After dislocating the hip, we found the femoral stem grossly loose and easy to remove. Joint fluid and tissue samples from the femoral canal were taken for analysis. Although the polyethylene acetabular cup showed minor eccentric wear and appeared excessively anteverted, it was firmly fixed. Hence, we decided to proceed with an isolated femoral revision.

We then removed excessive heterotopic ossification of the soft tissues posterior to the acetabulum to rotate the hip and access the femoral canal. Without trial stems and limited revision instrumentation, we prepared the femur, intending to bypass the area of cortical weakness by at least two shaft diameters. After make-shift trialing with the definitive implant and being reasonably satisfied, the femoral canal was rinsed and dried and the same femoral stem was cemented in place. Even with using the longest femoral neck, the hip remained completely unstable. We surmised that this was due to excessive anteversion and wear of the cup together with laxity of the soft tissues caused by removing the heterotopic ossification. Faced with this unanticipated problem, we chose to revise the acetabulum using a cemented all-polyethylene cup.

We proceeded to methodically remove the cup and cement, conscious of the underlying acetabular cage. The cage was stable, and 2 mm drill holes were made into the exposed bone to encourage cement interdigitation. An all-polyethylene cup was cemented in 40° abduction and 10° anteversion, and trial reduction indicated good stability using a 36 mm femoral head and an 8 mm neck. After inserting the final implants, we used a dilute povidone-iodine solution to soak the wound for three minutes. Before closure, using a layered water-tight approximation of the soft tissues, we conducted a thorough search for any bleeding and did not consider a drain necessary.

**Figure 5:**
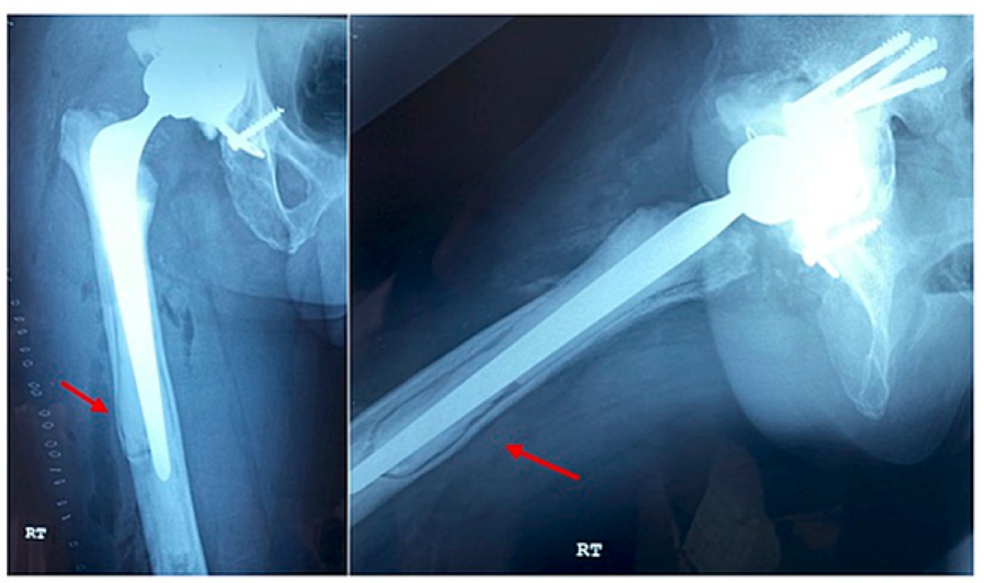
Postoperative radiographs showing a long-stem cemented femoral component. The red arrow indicates incomplete removal of the endosteal membrane at the level of maximum cortical osteolysis. Note that in the absence of a longer stem, the distal cement column has been extended to reduce the risk of a periprosthetic fracture.

Postoperatively, we placed the patient on intravenous antibiotics for five days (cefuroxime 1.5 g three times a day) and started oral anticoagulants (rivaroxaban 10 mg daily) 24 hours after the surgery. His postoperative X-rays were satisfactory, and mobilization started 24 hours after the surgery (Figure 5).

The patient’s postoperative hemoglobin was 9.8 g/dL which was well tolerated and did not affect his in-hospital rehabilitation. Two weeks post-surgery, the patient reported minimal pain and was progressing well with physiotherapy.

## Discussion

While revision THA in the JW patient population is associated with a higher rate of complications, studies have shown that it is safe under the correct circumstances. A successful outcome depends in part on the adoption of a comprehensive multimodal blood management protocol. As these protocols are well established in the developed world, it is not surprising that all six published studies describing the outcome of revision THA in JW patients have been conducted in the United States and the United Kingdom [6,7,11-14] (Table 1).

**Table 1:** Summary of reported revision THAs in JW. JW: Jehovah’s Witnesses; THA: total hip arthroplasty

Study	Year	Country	Number of patients	Mortality (%)
Nelson & Bowen [14]	1986	Arkansas, USA	24	0
Bonnett et al. [13]	1987	California, USA	2	0
Wittman & Wittman [12]	1994	Surrey, England	5	0
Sparling et al. [11]	1996	Arkansas, USA	5	0
Harwin et al. [6]	2012	New York, USA	10	0
Motla et al. [7]	2020	Washington, USA	5	0

In establishing an arthroplasty program in Trinidad, we are confronted with several unique challenges. Our antiquated blood donation system which relies on replacement donors for family or friends has led to a national undersupply of blood, and it is estimated that up to 70% of the country’s transfusion requirements remain unfulfilled [15]. In addition to this, the absence of a blood management protocol has resulted in a wide variability of transfusion practices following joint replacement [16]. The only study from the Caribbean that attempted to address this issue reported a 63% blood transfusion rate following primary arthroplasty [17]. Limited blood availability together with high transfusion rates has prompted a search for more innovative and cost-effective methods to reduce the need for blood during arthroplasty.

To our knowledge, this is the first report of a successful revision THA in the Caribbean, accomplished without the need for a blood transfusion. Notwithstanding our limited healthcare resources, we believe that bloodless arthroplasty can be effectively achieved with a restrictive transfusion protocol and implementation of an evidence-based blood management plan including (a) preoperative hemoglobin optimization, (b) hypotensive anesthesia, (c) meticulous surgical technique, and (d) the use of tranexamic acid (TXA).

Preoperative hemoglobin optimization

Preoperative hemoglobin optimization aims to eliminate sources of blood loss and maximize hemoglobin production capacity prior to surgery. This goal is supported by several studies which have shown a clear association between preoperative anemia and perioperative transfusion in patients undergoing hip and knee arthroplasty [18]. Three months prior to surgery all patients are screened in the clinic to identify any source of occult blood loss. Specifically, a history of melena, excessive use of non-steroid anti-inflammatory drugs, or aspirin use is identified. Women are asked about heavy menstruation or any post-menopausal bleeding. In this region, the use of herbal medicines is particularly common, several of which have the potential to interfere with hemostasis; therefore, it is of utmost importance that patients discontinue herbal medicines before surgery [19]. Iatrogenic blood loss is reduced by using pediatric phlebotomy tubes and avoiding unnecessary repeat blood tests [20]. We encourage natural erythropoiesis by placing all patients on oral supplementation of ferrous sulfate 325 mg thrice daily, vitamin C 500 mg twice daily, vitamin B12 1,000 mcg daily, and folic acid 1,000 mg daily [21].

Although recombinant human erythropoietin (rhEPO) effectively increases the concentration of hemoglobin and studies have demonstrated its safe use in JW patients undergoing joint replacement, it contains a small amount of albumin which may not be acceptable to some patients [11,22,23]. We have found that most JW patients accept rhEPO but cost is the major factor limiting its use in public hospitals.

Hypotensive anesthetic techniques

Hypotensive anesthesia effectively reduces blood loss during surgery by lowering the blood pressure to minimize intraoperative bleeding while maintaining perfusion to vital organs. The effectiveness of hypotensive anesthesia in reducing blood loss and transfusion in total hip replacement has been well established by many studies [14]. There are several modern methods to deliver hypotensive anesthesia, although traditionally, this was accomplished using inhalation techniques. In a 1986 study, Nelson et al. examined JW patients undergoing primary and revision THA using halothane and nitroprusside to provide hypotensive anesthesia and compared their outcomes to a control group. Compared with the control group, blood loss was reduced by 43% and 30% in the primary and revision THA groups [14]. In the rare case of a patient declining neuraxial anesthesia, as occurred in our index patient, we use either propofol infusion or inhalation with sevoflurane or isoflurane to achieve anesthesia. The blood pressure can then safely be adjusted with bolus doses of labetalol or hydralazine.

Neuraxial anesthesia is emerging as the preferred anesthetic technique in any contemporary arthroplasty service, and studies have demonstrated its safety in patients undergoing hip and knee arthroplasty [24]. We routinely use neuraxial anesthesia in our practice. Preoperatively, patients are encouraged to increase their oral intake in the days leading up to surgery, and clear fluids are allowed two hours before surgery to reduce the risk of dehydration and the need for intravenous fluids. Our technique involves a spinal anesthetic using heavy 0.5% bupivacaine without morphine for primary cases and an epidural catheter which is removed at the end of the operation for revision procedures. We aim to reduce the patients’ baseline mean arterial pressure by 30%, maintaining a steady systolic blood pressure between 60 and 80 mmHg, and avoid fluctuations that can lead to bleeding in uncoagulated vessels.

Preoperative planning and surgical technique

Preoperative surgical planning allows for a virtual rehearsal of the surgical steps and is an essential prerequisite for a successful outcome. The importance of this cognitive task is critical when undertaking complex and unpredictable procedures in which blood transfusion is not an option. In our resource-limited environment, preoperative planning takes on an even greater significance. The surgeon must ensure the availability of any special instruments required to facilitate exposure and removal of implants as an intraoperative delay can lead to unwanted blood loss. The variety of revision implants is restricted with a limited selection of available sizes, and the surgeon must be prepared to change the surgical strategy intraoperatively while maintaining the tempo of the operation. For example, although uncemented implants may be recommended, because of limited inventory, we commonly have to use cemented implants which allow for more intraoperative maneuverability.

As prolonged surgical duration increases blood loss, ensuring that the operation proceeds in a controlled and efficient manner is vital. Starting from the skin incision and proceeding to the deeper tissue dissection, meticulous hemostasis is maintained with the liberal but precise use of diathermy. Blood loss is minimized by gently handling the soft tissues and avoiding unnecessary stripping. During revision, removal of the implants (compounded by a femoral osteotomy) and reaming of the exposed bone are the two maneuvers that lead to the largest amount of blood loss [25]. To limit bleeding, gauze sponges are used to fill the dead space, effectively tamponading the exposed bony surfaces, similarly, they may be packed behind retractors and between fascial planes to minimize blood loss from the exposed soft tissues.

At the end of the procedure, we inject a periarticular local anesthetic cocktail (17.5 mL of 0.5% bupivacaine, 30 mg of ketorolac, 500 mcg of adrenaline, and 750 mg of cefuroxime made up to 100 mL with normal saline) into the soft tissues to help reduce bleeding. We do not use surgical drains; and when we are sure that the surgical field is dry, the wound is closed in layers using a locking suture. Thromboprophylaxis is provided by thromboembolic deterrent stockings and early mobilization, and aspirin (81 mg twice daily) is started 24 hours after surgery unless contraindicated [26].

Tranexamic acid

The use of TXA is supported by several studies that have reported less blood loss and fewer postoperative transfusions in both primary and revision joint arthroplasty [27-29]. Acting as an antifibrinolytic agent, TXA binds reversibly to the lysine-binding sites of plasminogen, inhibiting plasminogen from binding to fibrin, effectively reducing fibrinolysis [27]. Irrespective of its form (oral, topical, or intravenous), TXA has been shown to effectively lower blood loss following arthroplasty and is not associated with an increased risk of a postoperative thromboembolic event [27].

Our standard protocol for the administration of TXA is 1 g intravenously at the skin incision and another 1 g at closure. If the procedure is likely to last more than four hours, an additional 1 g is given at the two-hour mark [29]. In patients with contraindications to TXA, we use it topically as a soak at the end of the operation [30]. The use of TXA has revolutionized the practice of primary and revision arthroplasty and is now considered the standard of care. TXA is readily available in the public health sector and represents a low-cost, high-yield intervention that has been very effective in reducing the need for blood transfusion.

## Conclusions

Analyzing the outcome of a JW patient undergoing revision THA is of interest because it allows us to critically examine the steps used to reduce blood loss. Revision THA is regarded as a major reconstructive procedure that is associated with high rates of blood transfusion. Limiting the need for blood transfusion is a laudable goal for several reasons, including reduced complications and lower costs associated with the processing of blood, which is especially important in an underfunded health service. Finally, a major barrier to accessing arthroplasty services is removed, not only for JW patients but also for other patients discouraged from having this life-changing operation by the prospect of needing a blood transfusion. These principles have been used in our primary THA patients where the transfusion rate is <1%. We believe that wider use among arthroplasty surgeons will reduce the need for blood transfusion in patients undergoing a primary THA. Our blood management protocol for arthroplasty employs easily actionable steps that can be readily used without additional expense in a low-resource setting.
